# Neurosurgical residency: A lost cause or a last resort? Decoding the dilemma

**DOI:** 10.12669/pjms.40.12(PINS).11103

**Published:** 2024-12

**Authors:** Arooj Kiran, Sidra Ameer

**Affiliations:** 1 Dr. Arooj Kiran, MBBS Department of Neurosurgery, Punjab Institute of Neurosciences, Lahore, Pakistan; 2 Dr. Sidra Ameer, MBBS Kaul Associates (Department of Anaesthesia) Doctors Hospital and Medical Centre, Lahore, Pakistan

There is a universal rule called the 10,000 hours rule, which applies to any cognitively complex field, for example playing a violin or becoming a neurosurgeon. According to this rule, 10,000 hours of practice are required to excel in a competitive field. Neurosurgery, as a surgical specialty, is one of such fields hence, a neurosurgeon needs to be trained with finest skills, competencies and knowledge.^1^ Although the government has ensured to increase the number of post graduate slots and centers of neurosurgical training, yet we see a consistent decline in the quality of training and quantity of neurosurgical residents in Pakistan. Maintaining high quality training standards is a grueling task and here are some of the reasons why.

In 2016, to improve the quality of training and maintaining a transparent process of induction in the residency programs, the government introduced a policy famously known as central induction policy (CIP). This provided a valuable reference for tracking and analyzing the selection trends of candidates in different surgical disciplines across consecutive years. The statistics provided by the CIP clearly depicts that neurosurgery is still a low merit field as compared to the other specialties. In January 2024, the last merit of gynecology was 79.40%, dermatology 80.07%, radiology 78.21%, cardiology 74.93% as compared to the merit of 69.60% for neurosurgery indicating a relative lag in this specialty over the years.^2^ After closely observing this trend over the years, two things are important to note: why neurosurgery is a low merit field? What are its consequences?

Firstly, the demanding nature of neurosurgical training poses a significant challenge for aspiring surgeons. Compared to other specialties, neurosurgery demands a prolonged commitment of time and effort, the intensive workload, extended hours, and high-pressure environments may deter individual unwilling to endure such demanding conditions. Operating on the delicate structures of the brain and spine entails a high level of precision and carries inherent risks, including the potential for severe complications or patient mortality contributing to its status as a last specialty choice. In addition, neurosurgery is typically compensated financially less than or equal to the other medical specialties such as Dermatology and Ophthalmology, where there are defined working hours of approximately 36 hours per week, with fewer emergency responsibilities. Additionally, there are not enough centers in Pakistan to grapple with the high rate of neurosurgical trauma thus making the already existing centers overburdened and exhausted. Consequently, making the neurosurgery a low merit field in the healthcare pyramid.

Due to the booming number of private medical colleges in the past two decades, the number of new medical graduates have increased significantly, and this has raised the unemployment rate among the doctors. Everyday more and more doctors want to leave Pakistan according to a study by Nadir et al. in 2023, 33.3% doctors want to leave the country.^3^ However, these doctors need to pass some foreign licensing exam after their graduation to work abroad, to pass this time period and obtain valuable experience required for the foreign jobs, medical graduates, instead of opting for medical officer jobs or contract jobs, join residency programs in Pakistan. Neurosurgery is one of such unfortunate fields, frequently viewed as a transitional step by doctors, owing to its perceived lower merit and the availability of residency slots. Many individuals opt for neurosurgery residencies only to later pursue careers abroad in United States of America, United Kingdom, and Ireland. This trend results in a reduced number of residents in the field, placing additional strain on their colleagues.

Furthermore, the medical graduates are choosing it as a last resort to avoid unemployment due to the scarcity of the healthcare jobs in our country. Subsequently inducting more and more uninterested, unmotivated doctors in the training program, who eventually end up either leaving the training program midway due to exhaustion and overburden or failing their final examination due to lack of commitment and inspiration. Thus, leading to even further decline in the number of neurosurgeons in our country and the continuation of this vicious circle.

To combat the concerning decline in both the quantity and caliber of neurosurgical residents in Pakistan, a holistic approach is imperative. This involves not only enhancing work conditions and providing enticing financial incentives to elevate the allure of neurosurgery, but also expanding training facilities and resources, while actively engaging in awareness campaigns and mentorship programs. Moreover, reevaluating central induction policies and tackling the root causes of brain drain are paramount. Strictly implementing already existing measures like penalties or exit control to dissuade doctors from seeking opportunities abroad is crucial. This holistic strategy not only addresses the shortage of residents but also elevates the overall proficiency and resilience of the neurosurgical workforce, fostering a thriving future for the specialty in Pakistan.

## Authors Contribution

**AK** conceived, designed and wrote this manuscript, is responsible for integrity of this letter.

**SA** did data collection and manuscript writing.
